# Multifeature sequencing-based liquid biopsy for cancer diagnosis and monitoring

**DOI:** 10.1186/s13073-026-01739-2

**Published:** 2026-08-01

**Authors:** Mariano A. Molina, Martina De Simoni, Norbert Moldovan, Florent Mouliere, Daniel W. Hagey

**Affiliations:** 1 Department of Laboratory Medicine, Karolinska Institutet, ANA Futura, Huddinge, Sweden; 2 Department of Cellular Therapy and Allogeneic Stem Cell Transplantation (CAST), Karolinska University Hospital, Huddinge, Sweden; 3 Department of Pathology, Vrije Universiteit Amsterdam, Amsterdam UMC Location VUmc, Amsterdam, the Netherlands; 4 Faculty of Medicine, VU University Amsterdam, Amsterdam, The Netherlands; 5 Department of Medical Oncology, Erasmus MC, Rotterdam, The Netherlands; 6 Cancer Research UK National Biomarker Centre, University of Manchester, Manchester, UK

**Keywords:** Multifeature, Liquid biopsy, cfDNA, cfRNA, Microbial DNA

## Abstract

Liquid biopsy has emerged as a transformative development in oncology, enabling the minimally invasive detection and monitoring of cancer through the analysis of tumor-derived material in blood. Moving beyond single-variable analysis, multifeature sequencing-based liquid biopsy (MSLB) integrates diverse classes of data from a single blood sample to provide deep multifactorial insight into tumor biology. In this review, MSLB is defined as the extraction of multiple biological signals from a single sequencing dataset and is put in the context of other layers of multimodal diagnostics. We focus on how recent advances in patient-, and potentially microbe-derived, cell-free nucleic acid analysis expand the biological information that can be extracted from a single blood sample. MSLB enables this by allowing the concurrent assessment of, for example, DNA methylation, copy number, fragmentation, and, in exploratory workflows, microbe-associated signals. This provides a broader view of tumor, immune, and microenvironment states. When combined with emerging bioinformatic and machine-learning frameworks, these complementary signals may improve early detection, disease monitoring, and treatment selection. Addressing challenges in standardization, validation, and regulatory alignment will be essential to determine how MSLB can be integrated into routine oncologic practice.

**Supplementary Information:**

The online version contains supplementary material available at 10.1186/s13073-026-01739-2.

## Background

Liquid biopsy is a transformative concept for oncology, enabling the molecular characterization of cancer from minimally invasive blood samples [1–3]. Initially conceptualized for the identification of circulating tumor cells (CTC) in blood, the scope of liquid biopsy has since expanded to include the analysis of circulating cell-free proteins, lipids, metabolites, DNA (cfDNA), and RNA (cfRNA) [4–10]. This has enabled systemic and minimally invasive molecular monitoring of cancer, offering complementary information to tissue biopsy, which captures only a localized and often temporally limited view of tumor biology. Although liquid biopsies have their own biases, such as dependence on tumor shedding and selective representation of circulating clones, they can provide broader, longitudinal insights that are difficult to obtain from tissue alone [11–13].

Despite the diversity of potential targets, advances in next-generation sequencing technologies have been central to the evolution of liquid biopsy. For cfDNA, this has enabled highly sensitive detection of mutations, copy number alterations (CNAs), fragmentation patterns, and epigenetic aberrations [14–16]. Although not utilized clinically, the transcriptomic landscape of blood has also been found to provide insight into disease. As such, cfRNA-based sequencing has been applied to messenger (mRNA), micro (miRNA), circular (circRNAs), and long non-coding RNAs (lncRNA), among others [3, 17–19]. More recently, microbial DNA and RNA have been recognized as additional molecular components detectable within liquid biopsy datasets [20, 21]. The ability to interpret the quantity and diversity of biological information within these diverse targets represents the promise and challenge of liquid biopsy.

**Fig. 1:**
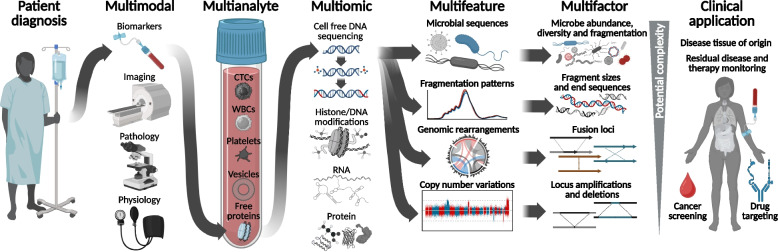
Multiple levels in sequencing-based liquid biopsy analyses. Multimodal analyses integrate complementary biological information derived from a blood sample, connecting clinical, imaging, and molecular data for comprehensive cancer profiling. In this framework, multianalyte approaches assess multiple circulating components from a single blood sample, such as circulating tumor cells (CTCs), white blood cells (WBCs), platelets, extracellular vesicles (EVs), and free proteins, to capture diverse biological signals. Multiomic approaches then interrogate multiple molecular layers, including genomic, transcriptomic, proteomic, lipidomic, and metabolomic profiles, within a single analyte. A single sequencing dataset can yield multiple complementary biological features through distinct bioinformatic workflows. From a single cell-free DNA library, diverse data layers can be extracted, including microbial sequences, fragment-size distributions, genomic rearrangements, and copy number alterations. Integrating these features enhances signal resolution and diagnostic accuracy without requiring additional experimental input. This level refers to multifeature sequencing-based liquid biopsy (MSLB), capturing orthogonal molecular information from the same dataset, bridging raw sequencing data and biological interpretation. Within each feature class, multifactor analyses can further assess multiple variables of the same type, such as alterations across multiple genomic loci or fragmentation metrics across genomic regions. Increasing analytical complexity across these levels may enhance signal resolution and diagnostic accuracy. This is an original figure created by the authors using BioRender (www.biorender.com)

Sequencing-based datasets are powerful because they can carry multifactorial genome-wide information, such as the expression levels of all genes. In contrast, clinical diagnosis relies on combining different single-variable inputs, such as biomarkers, imaging, pathology, and physiological data (multimodal analyses). These are generally assessed as normal or abnormal, rather than interpreted on a continuous, genome-wide scale (Additional File 1: Table S1) [22–24]. Liquid biopsy can leverage both strategies through multifeature sequencing-based liquid biopsy (MSLB). MSLB refers to the extraction of multiple, complementary molecular features from a single sequencing dataset spanning genomic, epigenomic, transcriptomic, fragmentomic, or microbial signals (Fig. 1). In most current implementations, MSLB refers to the integration of orthogonal patient-derived features, such as methylation, fragmentation, and copy number alterations, from a single sequencing library [25–27]. In contrast, microbe-associated analyses represent a controversial and exploratory extension of this framework, which requires increased sequencing depth, dedicated computational filtering, or specialized enrichment strategies. Since microbial nucleic acids typically comprise only a very small fraction of total circulating material, rigorous workflows are needed to incorporate this information [28–31].

At their highest level, multianalyte workflows have been developed to gain insight from different components of blood, such as CTCs and cfDNA, each of which has a distinct biological origin (Additional File 1: Table S1, Fig. 1). Multiomic approaches can then be applied to each analyte to capture distinct molecular information, such as by pairing genomic with transcriptomic analysis, which has been demonstrated to improve diagnostic and prognostic performance [32, 33]. Within a single sequencing data set, multifeature analysis can be deployed to capture distinct information using different bioinformatic workflows by, for instance, quantifying CNAs and fragmentomics from cfDNA sequencing (Fig. 1) [7, 34]. This distinction is important because many of these terms are inconsistently used in the liquid biopsy literature, creating conceptual ambiguity that this review aims to clarify.

Despite these technological advancements, the clinical adoption of MSLB remains limited, and most studies are never replicated in multiple centers [6, 35]. This is because the technical complexity of these approaches makes them sensitive to preanalytical conditions and difficult to standardize in diverse clinical settings [36, 37]. As such, current clinical practice continues to rely on single-variable assays, not because they are easier to interpret, but because they can be validated and certified under established quality-management frameworks far more readily than complex multifeature sequencing outputs. However, these simpler assays capture only a narrow portion of a cancer’s molecular diversity [34, 38, 39].

By using various strategies to extract complementary information from a minimally invasive assay, MSLB holds promise for enhancing diagnostic accuracy while simplifying workflows and reducing costs. Thus, we highlight recent advances in this area, examine their clinical relevance, and discuss how such integrative frameworks in multifeature analyses may catalyze the next phase of MSLB implementation in clinical oncology.

## Multifeature analysis by liquid biopsy analytes

### cfDNA

cfDNA comprises DNA fragments released into circulation, through cell death or exocytosis, which are referred to as circulating tumor DNA when originating from cancer cells [40]. cfDNA can harbor tumor-specific alterations and offers a minimally invasive window into the genetic landscape of cancer [41]. Mutation profiling has historically dominated sequencing-based cfDNA analysis, with panel strategies such as Guardant360 and FoundationOne Liquid CDx maturing into clinically applicable products in the US [42, 43]. More recently, the analytical scope of cfDNA has expanded to include epigenetic and structural features such as DNA methylation, nucleosome positioning, and fragmentomics [26, 44–46].

The cfDNA content in blood samples varies significantly by cancer type and stage, necessitating sensitive technologies often unavailable in clinical laboratories [47, 48]. High-shedding tumors such as liver, pancreatic, colorectal, and advanced lung cancers generally exhibit higher cfDNA fractions, whereas indolent tumors, certain pediatric cancers, and some early-stage malignancies may release substantially lower amounts of detectable cfDNA [49]. Of note, cfDNA shedding is influenced not only by tumor burden but also by biological factors such as tumor growth kinetics, cellular turnover, apoptosis, necrosis, vascularization, and anatomical site [50]. Mathematical modeling studies further suggest that cfDNA detectability is fundamentally constrained by shedding dynamics, blood sampling volume, sequencing error rates, and assay sensitivity, which together contribute to false-negative results in low-shedding cancers and early disease states [51]. To overcome these limitations, MSLB assays have emerged, capable of analyzing multiple molecular features from a single experimental workflow [7, 25, 26, 52]. While tumor-informed sequencing approaches currently dominate clinical applications focused on minimal residual disease (MRD) monitoring, MSLB has gained particular traction in the field of multi-cancer early detection (MCED), where feature-integrative designs offer greater diagnostic breadth across cancer types (Table 1) [46, 53].

**Table 1:** MSLB approaches using cfDNA

Technology	Features retrieved	Application	**Clinical use**	Approach	Notes	Reference
sWMS	CNAs, fragmentomics, methylation	Multi-cancer	MCED, tissue-of-origin	THEMIS	Enzymatic sequencing	[52]
WGS	CNAs, SNVs, methylation	Multi-cancer	MCED, MRD, symptomatic triage	TAPS	Deep (80x) sequencing	[53]
EM-seq	Methylation, nucleosome positioning, fragmentomics	CRC	ECD	MESA	Enzymatic sequencing	[26]
BS-seq	CNAs, global hypomethylation	Multi-cancer	ECD, MRD	NA	Bisulfite conversion	[54]
sWMS	CNAs, fragmentomics, methylation	Multi-cancer	MCED, tissue-of-origin	SPOT-MAS	Bisulfite conversion	[46, 55, 56]
IP-WMS	Methylation, fragmentomics, nucleosome footprints	Multi-cancer	MCED, tissue-of-origin	cfMeDIP-seq	MEDIPIPE pipeline	[57]
WGBS	CNAs, fragmentomics, methylation	ESCC	ECD	EMMA	Bisulfite conversion	[25]
sWGS	CNAs and fragmentomics	Glioma	ECD	NA	Applied to blood and urine	[58]
sWGS	CNAs, fragmentomics, nucleosome positioning, TF binding	Lung cancer	ECD, symptomatic triage	DELFI	Validated across multiple cancer types and cohorts	[59–61]
sWGS	CNAs and fragmentomics	Multi-cancer	MCED, recurrence monitoring	FrEIA	Multi-signal cfDNA integration	[7]
WGS	CNAs and fragmentomics	EwS	ECD, disease monitoring	LIQUORICE algorithm	Fragment coverage at cancer-specific open chromatin	[27]
WGS	CNAs, fragmentomics, nucleosome positioning	Multi-cancer	MCED	CANSCAN	Use of CNNs and GLMs; prediction of tissue-of-origin	[62]
WGS	CNAs, fragmentomics, nucleosome positioning, single nucleotide substitutions	Gastric cancer	ECD	NA	Two-layer ML classifier with Monte Carlo simulation for population screening modeling	[63]
WGS	Repeat element profiles (LINEs, SINEs, LTRs, satellites, TEs, RNA elements), fragmentomics	Multi-cancer	MCED, tissue-of-origin, disease monitoring	ARTEMIS	Alignment-free k-mer analysis; integrated with DELFI fragmentomics	[64]
WGS	Fragmentomics and inferred methylation	Breast and prostate cancers	Cancer detection, tissue-of-origin	FinaleMe	Non-homogeneous HMM predicting CpG-level methylation	[65]
WGS	Fragmentomics and inferred methylation	HCC and NPC	Cancer detection, tissue-of-origin	FRAGMA	Fragmentomics-based methylation inference	[16]
WGS	CNAs and fragmentomics	CRC	ECD	NA	Machine-learning classifier (SVM)	[66]
WGS	CNAs, nucleosome footprints, and inferred TF accessibility	CRC, prostate and breast cancers	ECD, tumor subtyping	NA	cfDNA-based inference of TF binding	[67]
ONT	CNAs, nucleosome positioning, and fragmentomics	Lung and bladder cancers	Disease monitoring	NA	Applied to blood and urine; < 24 hrs turnaround	[68]
ONT	CNAs, SNVs, fragmentomics	Esophageal and ovarian cancers	MRD, disease monitoring	nanoRCS	Monte Carlo-based tumor fraction estimation	[69]
Targeted sequencing	Exonic mutations, chromatin organization at TFs binding sites	SCLC	Tumor subtyping, disease monitoring	SCLCpheno-seq	Tumor-guided panel	[70]
Targeted sequencing	SNVs, CNAs, fragmentomics	NSCLC	ECD	Lung-CLiP	Tumor-naïve model integrating on- and off-target reads with machine learning	[71]

*ARTEMIS* Analysis of Repeat Elements in Disease, *BS-seq* Bisulfite Sequencing, *CNN* Convolutional Neural Network, *CNAs* Copy Number Alterations, *CRC* Colorectal Cancer, *DELFI* DNA Evaluation of Fragments for Early Interception, *ECD* Early Cancer Detection, *EMMA* Expanded Multimodal Analysis, *EM-seq* Enzymatic Methyl-seq, *ESCC* Esophageal Squamous Cell Carcinoma, *EwS* Ewing Sarcoma, *FinaleMe* Fragmentation Analysis of Cell-free DNA Methylation, *FrEIA* Fragment End Integrated Analysis, *FRAGMA* Fragmentomics-based Methylation Analysis, *GLM* Generalized Linear Model, *HCC* Hepatocellular Carcinoma, *HMM* Hidden Markov Model, *IP-WMS* Immunoprecipitation-enriched Whole Methylome Sequencing, *LINEs* Long Interspersed Nuclear Elements, *LIQUORICE* Liquid Biopsy Regions-of-interest Coverage Estimation, *Lung-CLiP* Lung Cancer Likelihood in Plasma, *LTRs* Long Terminal Repeats, *MCED* Multi-Cancer Early Detection, *MESA* Multimodal Epigenetic Sequencing Analysis, *ML* Machine Learning, *nanoRCS* Nanopore Rolling Circle Amplification-enhanced Consensus Sequencing, *NSCLC* Non–Small Cell Lung Cancer, *NPC* Nasopharyngeal Carcinoma, *ONT* Oxford Nanopore Technologies, *SCLC* Small Cell Lung Cancer, *SCLCpheno-seq* Small Cell Lung Cancer Phenotyping Sequencing, *SINEs* Short Interspersed Nuclear Elements, *SNVs* Single-Nucleotide Variants, *SPOT-MAS* Screening for the Presence Of Tumor by Methylation And Size, *SVM* Support Vector Machine, *sWGS* Shallow Whole-Genome Sequencing, *sWMS* Shallow Whole Methylome Sequencing, *TAPS* TET-Assisted Pyridine Borane Sequencing, *TEs* Transposable Elements, *TF* Transcription Factor, *THEMIS* Thorough Epigenetic Marker Integration Solution, *WGBS* Whole-Genome Bisulfite Sequencing, and *WGS* Whole-Genome Sequencing

DNA methylation profiling in cfDNA is commonly performed using bisulfite- or enzymatic-based conversion methods that distinguish methylated from unmethylated cytosines before sequencing. Traditional bisulfite sequencing techniques have been adapted for MSLB analyses, albeit with limitations [72]. These methods chemically degrade a substantial proportion of cfDNA molecules during bisulfite conversion, reducing the number of usable fragments for library preparation and consequently limiting the ability to recover CNAs and fragmentation-based features [73]. Nevertheless, a 2013 proof-of-concept study involving patients with multiple non-metastatic cancer types and healthy controls demonstrated the feasibility of co-detecting methylation and CNAs, with hypomethylation analysis achieving 74% sensitivity and 94% specificity for distinguishing cancer from non-cancer plasma samples [54]. More recently, Screening for the Presence of Tumor by Methylation and Size (SPOT-MAS) [46] and Expanded Multimodal Analysis (EMMA) [25] have applied bisulfite sequencing to integrate methylation, size distribution, and structural alterations. SPOT-MAS has demonstrated strong MCED performance across five tumor types, consistent with earlier colorectal and breast cancer-focused evaluations [55, 56], reporting sensitivities of ~ 72% and specificities of ~ 97% for cancer detection. In contrast, EMMA is designed for esophageal squamous cell carcinoma (ESCC) and assessed in patients with intraepithelial neoplasia, and matched healthy controls, achieving AUCs of 0.90–0.99 with 62% sensitivity at > 95% specificity in precancerous lesion detection (Table 1).

Among the most clinically advanced examples of methylation-based MCED is the Galleri assay developed through the Circulating Cell-free Genome Atlas (CCGA) study [74]. Using targeted bisulfite cfDNA methylation profiling and machine-learning classification, the assay demonstrated high specificity and accurate tissue-of-origin prediction across more than 50 cancer types [74]. Although these studies report strong performance, many were developed or validated within case–control or retrospectively assembled cohorts, which may overestimate diagnostic performance compared with population-based screening. In addition, bisulfite-induced DNA loss may limit the recovery of multifeature signals, constraining their applicability to MSLB workflows [25, 46].

Enzymatic sequencing-based methods have gained traction due to their ability to preserve the integrity of DNA modifications, thereby improving the concurrent analysis of multiple features [14, 75, 76]. One such example is the genome TET-Assisted Pyridine Borane Sequencing method (TAPS), which enables simultaneous profiling of methylation, somatic mutation burden, and CNAs from cfDNA via whole-genome sequencing using the TET2 enzyme and pyridine borane to selectively convert methylcytosines during processing [53]. This has been applied to plasma from patients diagnosed with six cancer types (colorectal, esophageal, pancreatic, renal, ovarian, and breast), achieving an overall sensitivity of ~ 95% and a specificity of ~ 89% for distinguishing cancer from non-cancer samples. The method was also explored for longitudinal disease monitoring and MRD assessment [53]. With a similar approach to TAPS, the Thorough Epigenetic Marker Integration Solution (THEMIS) [52] combines the detection of CNAs, fragmentomics, and methylation. In a study involving 1,277 plasma samples from healthy individuals and cancer patients across multiple tumor types (breast, colorectal, esophageal, gastric, liver, lung, and pancreatic), THEMIS achieved 73% sensitivity at 99% specificity for stage I-II cancers in the test cohort (Table 1) [52]. Although these approaches show considerable promise, their retrospective design and limited representation of early-stage cancers underscore the need for prospective validation in stratified cohorts.

Another enzymatic method, the Multimodal Epigenetic Sequencing Analysis (MESA) [26], was developed specifically for colorectal cancer detection. This method integrates targeted methylation profiling with cfDNA fragmentation metrics, such as nucleosome occupancy and window protection scores, and employs machine learning classifiers, including Random Forest, coupled with leave-one-out cross-validation (Table 1) [26]. Even though this method demonstrated strong cross-cohort performance, its design around known colorectal cancer-specific alterations makes it better suited for targeted screening in defined-risk populations rather than broad, tumor-agnostic MCED applications. Likewise, machine-learning analysis of cfDNA whole-genome sequencing profiles has also demonstrated promising performance for early-stage colorectal cancer detection, achieving a mean AUC of 0.92 in a cohort enriched for stage I-II disease through the integration of genome-wide fragmentation and CNAs [66]. More recently, cell-free methylated DNA immunoprecipitation and high-throughput sequencing (cfMeDIP-seq)-based workflows have also demonstrated that paired-end methylation-enriched sequencing libraries can simultaneously yield fragmentomic, nucleosome footprinting, and 5′ end motif information, enabling integrated MSLB from a single assay [57].

Beyond direct methylation sequencing approaches, computational frameworks such as FinaleMe have demonstrated that cfDNA fragmentation patterns derived from WGS can also be leveraged to infer methylation states and tissue-of-origin profiles without requiring bisulfite conversion of the analyzed cfDNA [65]. Similarly, the FRAGMA framework integrated genome-wide fragmentation patterns and methylation-associated signatures for cancer detection in hepatocellular and nasopharyngeal carcinoma patients and tissue-of-origin inference [16]. Together, these approaches highlight the growing convergence between fragmentomics, epigenetic inference, and MSLB analysis.

Whole-genome sequencing (WGS) and shallow WGS (sWGS) have also been leveraged for MSLB. Outside blood-based applications, sWGS integrating CNAs and fragmentomics has also been explored in cerebrospinal fluid (CSF) from glioma patients, enabling low-depth detection of tumor-derived cfDNA and molecular profiling without prior knowledge of tumor mutations [58]. A 2021 study also applied WGS to plasma cfDNA from Ewing sarcoma patients and other pediatric sarcomas [27]. It utilized meta-learning on chromatin-accessibility footprinting to develop a classifier for low-mutational burden cancers, achieving an AUC of 0.97 to distinguish Ewing sarcoma from healthy controls. More recently, a 2024 study integrated WGS-derived CNAs, fragmentomics, and single-nucleotide substitutions to distinguish stage I-II gastric cancer patients from non-cancer individuals, achieving sensitivities above 87% in two validation cohorts [63]. Moreover, Monte Carlo simulations demonstrated the feasibility of this approach for population-scale screening (Table 1). In line with this goal, the WGS-based CANSCAN test combines genetic and fragmentomic analysis for MCED [62]. Notably, CANSCAN uses a convolutional neural network (CNN) to model CNA features and generalized linear models and deep learning algorithms for multifeature classification. In an independent validation cohort including patients with cancer and non-cancer controls, the test achieved 87.4% sensitivity and 97.8% specificity for cancer detection. In a prospective study of 3,724 asymptomatic individuals, the test achieved 53.5% sensitivity, primarily detecting early-stage cancers, with 98.1% specificity [62]. Although further improvement is necessary for population-scale deployment, the large sample size and independent validation of this work support the potential of MSLB for MCED.

Another prominent example is the DNA Evaluation of Fragments for early Interception (DELFI) framework, which uses sWGS and machine learning to analyze genome-wide cfDNA fragmentation profiles for lung cancer detection and symptomatic patient triage [40, 59]. Similarly, the Fragment End Integrated Analysis (FrEIA)-based framework integrates genome-wide fragmentomic features, including fragment-end sequence patterns and size distributions, for cancer detection across multiple tumor types and for prognostic stratification and recurrence monitoring in selected cohorts [7]. More recently, the joint ARTEMIS-DELFI framework combined genome-wide repeat element landscape profiling with fragmentomic analysis to support cancer detection, tissue-of-origin classification, and longitudinal disease monitoring through cfDNA analysis [64]. The Lung Cancer Likelihood in Plasma (Lung-CLiP) approach integrates mutation, CNAs, and mutation-associated fragmentomic features through machine learning for the early detection of non-small cell lung cancer (NSCLC) [71]. Developed using the CAPP-Seq hybrid-capture platform, the study introduced duplex barcoding and optimized molecular recovery to improve variant detection. In a prospective independent validation cohort, the model achieved AUCs of 0.69, 0.71, and 0.98 for detection of stage I-III NSCLC, which is comparable to tumor-informed assays without requiring prior tumor sequencing (Table 1).

Recent advances in long-read and nanopore sequencing have further expanded the scope of cfDNA-based MSLB. Proof-of-concept studies in lung and bladder cancer demonstrated the feasibility of rapid nanopore-based multifeature cfDNA profiling, including CNAs, fragmentomics, and nucleosome-positioning profiles, supporting the potential of same-day liquid biopsy analysis in future clinical settings [68]. Likewise, NanoRCS was primarily developed for MSLB with potential applications in treatment monitoring and MRD detection, integrating SNVs, CNAs, and fragmentomics through rapid nanopore-based cfDNA sequencing in esophageal and ovarian malignancies [69].

Finally, novel MSLB strategies have integrated chromatin architecture and transcription factor binding site analysis into cfDNA workflows. For instance, Small Cell Lung Cancer Phenotyping Sequencing [70], introduced in 2024, combined targeted mutation detection, nucleosome profiling, and transcription factor occupancy mapping to distinguish small cell from NSCLC (Table 1). Similarly, transcription factor accessibility profiling from cfDNA fragmentation patterns has been applied to colorectal, prostate, and breast cancers, enabling early cancer detection as well as clinically relevant tumor subtyping, including the identification of neuroendocrine prostate cancer lineage states [67]. Although early data are promising, broader validation is needed to confirm the diagnostic utility of this approach across cancer types.

### cfRNA

cfRNA is released into circulation by both normal and malignant cells and represents a promising liquid biopsy target [77, 78]. cfRNA derived directly from tumor cells is referred to as circulating tumor RNA, and offers unique insights into the transcriptional landscape and regulatory states of cancer [50]. Although cfRNA might be abundant, mRNA is only estimated to comprise less than 5% of the total, with the remainder dominated by ribosomal and short RNAs [50]. This is because mRNA is fragmented during cell death, while unprotected cfRNA is rapidly degraded by RNases [79]. While this can pose a challenge to its analysis, extracellular vesicles (EV) are released by all cells and can protect a subset of cfRNAs from degradation [80]. Thus, cfRNA exists in two main forms: freely circulating RNA fragments and RNA packaged within extracellular vesicles (EV-cfRNA). These compartments differ in stability and RNA composition. Together, they provide complementary access to coding and non-coding transcripts. Importantly, EV-cfRNA has been shown to reflect disease state and carry pathogenic alterations in its mRNA [81]. As such, cfRNA holds the potential to deliver distinct multifeatured biological information based on the abundance, sequences, fragment sizes, and post-transcriptional modifications of coding and non-coding RNA species [78, 82, 83].

For instance, Phospho-RNA-seq was designed to recover and characterize fragmented mRNAs and lncRNAs from plasma using a rigorous bioinformatic pipeline to enhance the fidelity of transcriptome reconstruction [84]. However, such methods still need to be validated against quantitative measures and face challenges related to highly abundant RNA species such as ribosomal and Y RNAs, which still dominate sequencing libraries (Table 2). To address this, Polyadenylation Ligation-Mediated Sequencing (PALM-Seq) employs RNAse H-based depletion of abundant RNAs coupled with iterative read alignment to profile a broad spectrum of RNA classes, ranging from miRNAs and piRNAs to tRNAs, mRNAs, and lncRNAs [85]. In addition to enabling broad transcriptomic coverage, this allows RNA fragmentomics to be analyzed as a unique data feature. Most recently, Random priming and Affinity capture of cfRNA fragments for Enrichment analysis by sequencing (RARE-seq) combines analyses of gene expression, splicing, fusion, mutation, and tissue-origin features and was demonstrated to be 50-fold more sensitive than whole-transcriptome RNA-seq [86]. Applied to over 400 plasma samples, it enabled tumor-naïve detection of NSCLC with up to 83% sensitivity in stage IV disease, genotyping of actionable variants, and identification of resistance mechanisms to EGFR inhibitors, including histological transformation. The method establishes cfRNA as a powerful, multi-dimensional biomarker complementing cfDNA-based liquid biopsy (Table 2) [86]. However, the high biological variability of cfRNA and sensitivity to preanalytical handling still limit its deployment outside controlled research settings.

**Table 2:** Potential MSLB approaches using cell-free host and/or microbial RNA

Technology	Features retrieved	Application	Clinical use	Approach	Notes	Reference
Enzymatic small RNA-seq	Host mRNA, lncRNA, miRNA	Plasma cfRNA profiling	NA	Phospho-RNA-seq	RNA pretreatment with T4 polynucleotide kinase	[84]
Enzymatic RNA-seq	Host mRNA, lncRNA, sRNAs, fragmentomics	Multi-biofluid cfRNA profiling	NA	PALM-Seq	No size selection, low-input compatible	[85]
Targeted RNA-seq	Host mRNA, somatic variants	Multi-cancer	ECD, tissue-of-origin, disease monitoring	RARE-seq	Hybrid capture targeting RAGs	[86]
Small RNA-seq	Host and bacterial sRNAs	Multi-biofluid sRNA profiling	NA	sMETASeq	Computational pipeline; SAA	[87]
Small RNA-seq	Host and bacterial sRNA	Multi-biofluid sRNA profiling	NA	sRNAflow	Computational pipeline; SAA and CAA	[88]
RNA-seq	Host mRNA and sRNA, bacterial RNA	NSCLC	ECD	cfRNA-Seq	Computational pipeline; SAA	[89]
Enzymatic small RNA-seq	Host RNA modifications, sRNA, and fragmentomics, bacterial RNA and inferred methylation	CRC	ECD	LIME-seq	Machine-learning classifier (SVM); SAA	[21]
RNA-seq	Host mRNA, lncRNA, sRNA, bacterial and viral RNA	Multi-cancer	MCED, tissue-of-origin	SMART-total	CRISPR-guided rRNA depletion; SAA	[30]
RNA-seq	Host mRNA, lncRNA, sRNA, bacterial RNA	Multi-cancer	MCED, tissue-of-origin	DETECTOR-seq	CRISPR-guided rRNA depletion; SAA	[90]

*CAA* Concurrent Analytical Alignment, *cfRNA* Cell-Free RNA, *cfRNA-Seq* Cell-Free RNA Sequencing, *CRC* Colorectal Cancer, *CRISPR* Clustered Regularly Interspaced Short Palindromic Repeats, *ECD* Early Cancer Detection, *lncRNA* Long Non-Coding RNA, *LIME-seq* Low-Input Multiple Methylation Sequencing, *MCED* Multi-Cancer Early Detection, *miRNA* MicroRNA, *mRNA* Messenger RNA, *NA* Not applicable, *NSCLC* Non–Small Cell Lung Cancer, *PALM-Seq* Polyadenylation Ligation-Mediated Sequencing, *RAGs* Rare Abundance Genes, *RARE-seq* Random Priming and Affinity Capture RNA Enrichment Sequencing, *rRNA* Ribosomal RNA, *sMETASeq* Small-RNA Metagenomics by Sequencing, *SMART-total* SMART-Based Total RNA Sequencing, *sRNA* Small RNA, *sRNAflow* Small RNA Flow Pipeline, *SAA* Stepwise Analytical Alignment, *tRNA* Transfer RNA

Finally, multifeature cfRNA can also be applied directly to cancer cells, as with Digital Microfluidics-Enabled Dual-Modal sequencing (DMF-DM-seq) [91]. This enables the co-profiling of mRNAs and miRNAs at the single-cell level through a microfluidic platform, which might be useful for high-throughput MSLB analyses of CTCs. Together, these studies demonstrate the diverse RNA analytes, molecular targets, features, and factors that can potentially be exploited by MSLB strategies.

### Circulating microbial nucleic acids

Unlike patient-derived cfDNA and cfRNA, the interpretation of circulating microbial nucleic acids remains highly controversial [92, 93]. Because microbial biomass in blood is extremely low, sequencing-based analyses are particularly susceptible to environmental contamination introduced during sample collection, extraction, library preparation, or computational processing [31, 94]. Several studies reporting cancer-associated microbial signatures have therefore faced criticism regarding contamination control and biological interpretation, including the re-evaluation and retraction of influential plasma metagenomic datasets [92, 95]. Furthermore, it remains unclear to what extent many tissue-associated microbes release detectable nucleic acids into circulation at quantities sufficient for robust clinical testing. Consequently, microbial-derived circulating signals should currently be considered emerging biomarker layers within MSLB frameworks and not as clinically validated biomarkers for most cancers. Nevertheless, integration of microbial-derived features with host-derived molecular signals holds the potential to improve cancer detection, tissue-of-origin prediction, and biological interpretation in future MSLB approaches.

Despite these challenges, recent developments have expanded cfDNA and cfRNA analyses beyond patient-derived signals to include circulating microbial DNA (cmDNA) and RNA (cmRNA) [20, 96]. In many cases, these microbial-derived signals are computationally extracted from broader cfDNA or cfRNA sequencing datasets generated by patient-derived sequence analyses [86, 97]. This positions them conceptually within the MSLB framework, despite requiring metagenomic approaches accounting for contamination and analytical bias to uncover the microbial signatures present in blood [94]. As a result, reliable detection and annotation of cmDNA and cmRNA remain major technical challenges and active areas of methodological development.

Unlike the gut and skin, increasing evidence suggests that healthy individuals lack a genuine active microbiome in the blood [31]. Consequently, the presence of microbial nucleic acids in circulation may signal underlying pathology, such as sepsis or malignancy [98]. As such, recent studies have identified distinct resident microbes and microbial signatures associated with various cancer types and clinical outcomes [99, 100]. For instance, cmDNA and cmRNA profiles have been proposed for the detection of hepatocellular carcinoma [96], lung cancer [28, 97], subtyping of myeloid malignancies [29], and predicting therapeutic response in colorectal cancer [101]. These findings suggest that microbe-associated signals may have the potential to complement patient-derived features in some MSLB workflows, although substantial technical and biological uncertainties remain.

Several recent bioinformatics tools have been developed to facilitate multifeature profiling of host and microbial RNAs from a single sequencing dataset. These most often employ a stepwise analytical alignment (SAA) strategy: sequencing reads are first aligned to the human genome to identify endogenous RNAs, and the remaining unmapped reads are subsequently interrogated for their microbial origin. However, this stepwise approach may lead to false negatives or misclassifications for conserved sequences, due to overly strict initial human mapping or sequence overlap between humans and microbes. Alternatively, a concurrent analytical alignment (CAA) to human and microbial reference genomes can reduce the risk of ambiguous or incorrect assignments and improve the accuracy of microbial sequence profiling [102].

For example, Small-RNA Metagenomics by Sequencing (sMETASeq) utilizes Kraken [103, 104] for taxonomic classification and has been evaluated across a diverse array of samples, including tissue specimens, bodily fluids, and a synthetic microbial community (Table 2) [87]. In contrast, sRNAflow introduces a hybrid, alignment and clustering-based, small RNA identification strategy that simultaneously queries both host and microbial reference databases [88]. In a proof-of-concept study, sRNAflow was applied to simulated human and microbial datasets, achieving 99% specificity and sensitivity for RNA-source assignment [105]. A third tool, sRNAbench-microbes, was recently built on the sRNAtoolbox suite that was initially developed for contamination screening [106]. As such, its utility in MSLB remains untested, underscoring the many potential sources of progress in this area and the need for validation in clinically relevant settings.

Towards this goal, cfRNA-seq is a recently developed multifeature pipeline designed for the early detection of NSCLC [89]. This method jointly analyzes coding, non-coding, immune-related, and microbial RNA features from cfRNA, and a classifier based on gene-expression signatures achieved an AUC of 0.9 in distinguishing cancer from non-cancer samples. Importantly, it also revealed cancer-associated microbial signatures and allowed for immune repertoire reconstruction (Table 2) [89]. To assess additional features, Low-Input multiple Methylation sequencing (LIME-seq) utilizes a recombinant reverse transcriptase to detect RNA abundance and methylation signatures for microbial taxonomic classification [21]. Using an SAA workflow and Kraken2, this used microbial RNA modification patterns to enable highly accurate classification of colorectal cancer patients vs non-cancer controls (AUC = 0.98) [21]. When comparing microbial and human RNA signatures, another study found these to achieve MCED to a similar degree (AUC 0.82 and 0.91, respectively), with liver and esophageal cancers best detected in cmRNA (Table 2) [30]. Furthermore, the same authors reported Depletion-assisted multiplexed cell-free total RNA sequencing (DETECTOR-seq), and compared the diagnostic performance of profiling whole plasma and isolated EVs [90]. This strategy integrates early UMI barcoding and CRISPR-Cas9-based rRNA/mtRNA depletion and found that different analytes performed best depending on the genes analyzed. Interestingly, both of these reports found that cmRNA signatures were better able to distinguish different types of cancer from one another than those from human-derived cfRNA signatures [92]. Notably, the authors also recovered viral signatures in blood [30], which have previously been described primarily for circulating human papillomavirus (HPV) DNA in cervical and oropharyngeal cancer patients [107, 108]. Likewise, plasma Epstein-Barr Virus (EBV) DNA is already integrated into screening and monitoring workflows for nasopharyngeal carcinoma in several regions, demonstrating that microbial-derived nucleic acids can achieve clinical utility when strong biological linkage, high shedding, and standardized analytical frameworks are present [109, 110].

To understand the source of microbial sequences in blood, growing attention has been directed toward bacterial extracellular vesicles (BEV) as potential carriers of cmDNA and cmRNA [111]. BEVs are membrane-bound nanostructures actively secreted by bacteria, which can cross epithelial barriers to enter systemic circulation [112]. Once in the bloodstream, BEVs may interact with host immune cells or tumor microenvironments, influencing inflammation, immune modulation, and cancer progression [112, 113]. As with human EVs, BEVs carry and protect a variety of biomolecules, including lipids, proteins, DNA, and RNAs. This makes them a potential source of cmDNA and cmRNA, although direct evidence linking BEVs to cancer-associated plasma microbial signatures remains limited [114–116]. Various studies have identified distinct BEV signatures in colorectal cancer tissue [115] and in the blood of breast, ovarian, and endometrial cancer patients [117]. BEVs therefore represent a potential biological mediator explaining microbial signals captured within MSLB pipelines. However, distinguishing BEV-derived signals from other sources and environmental contamination remains a technical challenge [111, 113]. Microbe-associated MSLB assays may ultimately prove most useful in cancers with strong microbiome associations, such as colorectal, gastrointestinal, or HPV-associated malignancies, or in contexts where microbial-host interactions influence therapeutic response [118–120]. In these settings, integrating BEV profiling with host-derived molecular signatures could potentially improve cancer detection, biological interpretation, and disease monitoring. However, the role of microbe-derived signals in screening, diagnosis, and longitudinal monitoring remains uncertain and will require rigorous prospective validation.

## Clinical applications of multifeature liquid biopsy analysis

**Fig. 2:**
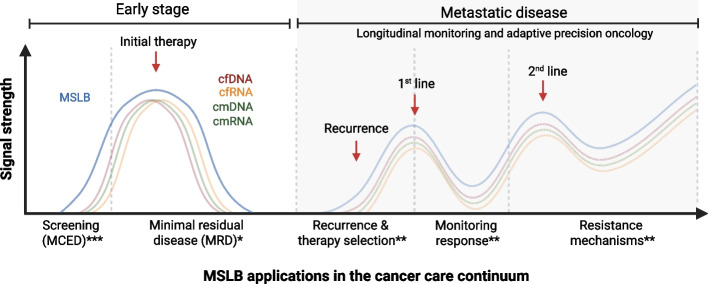
Potential clinical applications of MSLB across the cancer care continuum. This schematic illustrates a theoretical pattern of how different circulating analytes could contribute to MSLB features at various stages of cancer. MSLB approaches are mostly applicable for MCED and disease characterization and may have potential for other applications in the cancer care continuum. During early cancer stages, cfDNA (red), cfRNA (yellow), microbial DNA/RNA (cmDNA/RNA) (green), or their combined signal (cfDNA + cmDNA, or cfRNA + cmRNA, blue) may be detected to varying degrees. After initial therapy (e.g., surgery, chemotherapy, or radiotherapy), these signals decline but may re-emerge (dashed lines) during recurrence, therapy monitoring, and resistance detection. The shaded region denotes the phase of longitudinal monitoring and adaptive precision oncology, encompassing first-line (1st line) and second-line (2nd line) systemic therapies, where MSLB could potentially support dynamic tracking of tumor evolution, treatment response, and resistance mechanisms through multifeature analysis of cfDNA, cfRNA, and microbial signals within unified bioinformatic and machine-learning pipelines (combined signal, blue). Asterisks indicate the relative potential utility of MSLB approaches across clinical applications, with *** indicating higher potential utility, ** moderate or emerging utility, and * settings where untargeted MSLB approaches may face analytical sensitivity limitations. This is an original figure created by the authors using BioRender (www.biorender.com)

MSLB analyses are redefining the landscape of cancer diagnostics and monitoring by integrating multiple layers of molecular information, such as genomic, epigenomic, transcriptomic, and metagenomic data, from a single blood sample. This integrative approach addresses key limitations of traditional diagnostic approaches, including low sensitivity, high cost, and the time needed for patients to undergo multiple procedures. By enhancing the signal-to-noise ratio and reducing diagnostic subjectivity, multifeature workflows can detect subtle physiological changes that may elude single-variable methods [34]. These advances may be particularly valuable for MCED, as they have the potential to enhance both sensitivity and specificity in a minimally invasive and repeatable assay (Fig. 2).

Clinically, each modality within MSLB offers distinct advantages and limitations. Mutation-based assays provide high specificity but suffer from limited sensitivity in early-stage and low-shedding tumors [121, 122]. CNA and fragmentomics approaches are tumor-agnostic and scalable but lack adequate genomic resolution. Methylation profiling increases sensitivity and tissue-of-origin resolution yet requires complex workflows and stringent quality control [123]. cfRNA adds real-time information on transcriptional and immune activity but remains technically variable and challenging to standardize [124]. Microbe-derived signals may capture microenvironmental or host-tumor interactions, but their biological interpretation remains controversial [113]. Integrating these modalities through MSLB is therefore clinically attractive, as each feature has the potential to contribute distinct information across the cancer care continuum, but remains challenging in practice (Fig. 2).

Cost-effectiveness is an important consideration for clinical implementation. Sequencing-intensive assays require specialized infrastructure, bioinformatic expertise, and standardized computational workflows, which may limit accessibility outside centralized centers [125]. Consequently, MSLB may not be appropriate for all patients or clinical contexts. Instead, these approaches may initially provide the greatest value in high-risk populations, cancers lacking effective screening strategies, cancers of unknown primary, or longitudinal monitoring settings where repeated tissue biopsies are impractical [126]. Moreover, the optimal MSLB design will likely differ according to clinical application, as broad MCED strategies, tumor-informed MRD assays, and therapy-selection workflows each impose distinct requirements for sensitivity, specificity, turnaround time, and cost [127]. However, set against these limitations are the continuously decreasing costs of sequencing and processing power, which drive investment towards the promise of MSLB approaches.

Commercial MCED assays such as Galleri [74, 128] and Cancerguard [129] primarily rely on a single dominant molecular modality, most commonly cfDNA methylation, which constrains biological breadth and can limit sensitivity for low-shedding tumors. Although these approaches have demonstrated strong performance in large cohorts, they interrogate only one dimension of the circulating tumor signal [74, 128, 129]. In contrast, MSLB integrates multiple orthogonal features from the same sample, enabling broader molecular coverage and potentially more robust cancer detection and tissue classification. The rationale for MSLB is not that each feature independently achieves optimal sensitivity, but that partially orthogonal biological signals may compensate for one another across heterogeneous tumors and disease states. For example, tumors with limited mutational shedding may still exhibit detectable methylation abnormalities or fragmentation changes. Integrative machine-learning frameworks can therefore combine weak but complementary signals to improve overall classification performance, tissue-of-origin prediction, and robustness across patient populations. This principle underlies many current MSLB frameworks, including Random Forest-, SVM-, CNN-, HMM-, and deep learning-based classifiers that integrate fragmentomic, methylation, transcriptional, and genomic features from single sequencing datasets (Tables 1 and 2) [26, 62, 65, 66]. However, applications requiring extremely low limits of detection (LOD), such as MRD assessment, may still favor tumor-informed or highly targeted assays in some clinical contexts, as broad untargeted multifeature workflows can face analytical sensitivity limitations at very low cfDNA fractions. Nevertheless, several recent MSLB approaches have demonstrated promising applications in longitudinal disease monitoring and MRD detection (Tables 1 and 2, Fig. 2) [53, 54, 69].

Despite this promise, detecting disease exclusively through molecular signals introduces a series of practical and ethical uncertainties. When an MSLB assay yields strong evidence of malignancy in the absence of radiologically detectable lesions, clinicians may face difficult decisions regarding follow-up, intervention, and communication with patients [36]. Even highly specific assays may identify biological changes long before macroscopic disease becomes visible, potentially creating prolonged periods of uncertainty and psychological burden. Importantly, it remains unknown whether initiating therapy at this molecular stage improves outcomes compared with continued surveillance, as prospective randomized evidence is still lacking [130]. These uncertainties are particularly relevant for MRD monitoring, where the risk of overtreatment must be carefully weighed against relapse prevention [131]. MSLB may therefore improve confidence in truly tumor-derived signals and help distinguish clinically meaningful biology from transient or low-level noise. Moreover, multifeature signatures may provide insight into tumor hallmarks, aggressiveness, or evolutionary trajectories, which could one day inform whether a molecular signal warrants immediate intervention or closer longitudinal surveillance (Fig. 2).

Beyond detection and surveillance, MSLB provides a route to deeper biological interpretation of tumors. By combining host and microbial features within a unified framework, MSLB can begin to resolve which oncogenic pathways, such as genomic instability, immune evasion, metabolic reprogramming, or proliferative signaling, are most active in a given patient. Such integrative profiling has the potential to inform therapeutic selection (Fig. 2), particularly in settings where tissue biopsies are limited, unrepresentative, or infeasible. For example, integrated cfDNA methylation, fragmentation, and mutation profiling may improve molecular classification of lung cancers, including identification of neuroendocrine transformation states associated with resistance to EGFR-targeted therapies [70]. Similarly, cfRNA MSLB liquid biopsy approaches have been explored to identify actionable resistance mechanisms and longitudinal treatment response in NSCLC and gastrointestinal malignancies [86]. These strategies may be particularly valuable for patients in whom tissue biopsy is insufficient or unlikely to capture spatial and temporal tumor heterogeneity, such as metastatic disease or longitudinal monitoring during systemic therapy. However, the clinical utility of these approaches for therapy selection remains largely investigational and requires prospective validation. Leveraging these signatures for biological interpretation, therefore, represents an emerging frontier in precision oncology and may help bridge the gap between molecular detection and clinically actionable decision-making.

Still, several barriers hinder the widespread clinical adoption of MSLB assays. The field currently lacks universally accepted gold standards and reference controls for the analysis of multiple features in cfDNA and cfRNA [132]. These will be required for the external validation of individual feature detection, which otherwise complicates cross-platform comparison and limits approval by regulatory agencies. Moreover, the sensitivity of these methods and reliance on machine learning make their results vulnerable to technical variability and batch effects. These include differences in sample processing, sequencing depth, library preparation, and bioinformatics pipelines, which are difficult to control between healthcare centers [124]. In addition, biological differences between populations, variability in standard-of-care practices, sequencing platforms, and preanalytical handling may all influence assay performance and limit cross-cohort reproducibility. This contributes to a fear of false-negative and false-positive results, particularly for low-shedding tumors and population-level screening applications, where misdiagnosis can permanently harm confidence in MSLB approaches overall [133].

In the near term, clinical implementation of MSLB will likely occur first in clinically enriched settings instead of population-wide screening programs [134]. Applications such as longitudinal disease monitoring, treatment response assessment, cancers lacking effective screening strategies, cancers of unknown primary, or situations where repeated tissue biopsies are impractical may represent the most immediate translational opportunities. Broad MCED applications will require large prospective multicenter validation studies across diverse populations to establish clinical utility, reproducibility, and cost-effectiveness [135]. Thus, future implementation will also require standardized preanalytical workflows, external benchmarking datasets, clinically interpretable machine-learning models, and regulatory frameworks capable of evaluating multifeature assays [136].

Foundation models could have the potential to bypass some of these limitations if trained on sufficiently large and diverse cohorts [137]. Recent studies have begun exploring large-scale machine-learning and foundation-model frameworks trained on cfDNA fragmentomics, methylation, and cfRNA datasets to improve cancer classification and biomarker discovery [27, 55, 59, 60, 66, 74]. These approaches may facilitate transfer learning across cancer types and sequencing platforms, with generalizable patterns allowing integration of highly complex multifeature datasets. However, the clinical implementation of such models will require external validation and transparency regarding model interpretability and training bias [136]. Thus, rigorous controls, standardized pipelines, and prospective validation in diverse cohorts are urgently needed to translate these approaches into clinical practice [113].

## Conclusions and future perspectives

MSLB represents a conceptual shift toward integrative, genome-scale interpretation of liquid biopsy data in oncology, offering the ability to decode complex tumor biology from minimally invasive samples by integrating layers of complementary molecular information. Despite its promise, widespread clinical adoption of MSLB has been slow. Technical, logistical, and regulatory hurdles, including assay standardization, cost, and bioinformatic interpretability, must be addressed to achieve broad implementation. In particular, the dynamic nature of blood and the sensitivity of multifeature assays make the standardization of preanalytical conditions and sample workflows essential. Despite this, MSLB holds considerable potential to increase diagnostic sensitivity and specificity for complex and diverse diseases, such as cancer, for several reasons. First, by accessing a large amount of information, unbiased and global analyses are a key development in diagnostics. Second, by targeting multiple biological features, MSLB can access complementary aspects of disease in a single, repeatable assay. Finally, the economics and scalability of sequencing-based methods are significant advantages over mass spectrometry and array-based platforms. This puts particular focus on the importance of research into circulating nucleic acids, in parallel with advances in sequencing technologies [16, 138–142], computational biology tools [142–145], CRISPR-Cas targeting [146–150], artificial intelligence [137, 151], and microfluidic platforms [152, 153]. Together, these fields have the potential to fundamentally alter disease diagnosis and management.

Future progress will depend not simply on generating more molecular data, but on determining which combinations of complementary features provide clinically meaningful information beyond established single-feature assays. MSLB offers a framework to extract genomic, epigenomic, fragmentomic, transcriptomic, and microbial signals from the same dataset, maximizing the biological information obtained from limited circulating material. The next challenge is therefore to translate this analytical breadth into reproducible and clinically interpretable signatures tailored to specific applications, from early detection to longitudinal monitoring and therapy selection. If supported by prospective multicenter validation and standardized analytical frameworks, MSLB may shift liquid biopsy from the detection of isolated molecular abnormalities toward an integrated and dynamic representation of cancer biology.

## Supplementary Information

Additional file 1.

## Data Availability

No datasets were generated or analysed during the current study.

## Abbreviations

ARTEMIS: Analysis of repeat elements in disease
AUC: Area under the curve
BEVs: Bacterial extracellular vesicles
BS-seq: Bisulfite sequencing
CAA: Concurrent analytical alignment
CAPP-Seq: Cancer personalized profiling by deep sequencing
CCGA: Circulating cell-free genome atlas
cfDNA: Cell-free DNA
cfMeDIP-seq: Cell-free methylated DNA immunoprecipitation and high-throughput sequencing
cfRNA: Cell-free RNA
cfRNA-seq: Cell-free RNA sequencing
circRNAs: Circular RNAs
cmDNA: Circulating microbial DNA
cmRNA: Circulating microbial RNA
CNAs: Copy number alterations
CNN: Convolutional neural network
CpG: Cytosine-phosphate-guanine
CRC: Colorectal cancer
CRISPR: Clustered regularly interspaced short palindromic repeats
CSF: Cerebrospinal fluid
CTC: Circulating tumor cell
DELFI: DNA evaluation of fragments for early interception
DETECTOR-seq: Depletion-assisted multiplexed cell-free total RNA sequencing
DMF-DM-seq: Digital microfluidics-enabled dual-modal sequencing
EBV: Epstein-Barr virus
ECD: Early cancer detection
EGFR: Epidermal growth factor receptor
EMMA: Expanded multimodal analysis
EM-seq: Enzymatic methyl sequencing
ESCC: Esophageal squamous cell carcinoma
EV: Extracellular vesicle
EV-cfRNA: Extracellular vesicle-associated cell-free RNA
EwS: Ewing sarcoma
FinaleMe: Fragmentation analysis of cell-free DNA methylation
FrEIA: Fragment end integrated analysis
FRAGMA: Fragmentomics-based methylation analysis
GLM: Generalized linear model
HCC: Hepatocellular carcinoma
HMM: Hidden Markov model
HPV: Human papillomavirus
IP-WMS: Immunoprecipitation-enriched whole methylome sequencing
LINEs: Long interspersed nuclear elements
LIQUORICE: Liquid biopsy regions-of-interest coverage estimation
LIME-seq: Low-input multiple methylation sequencing
lncRNA: Long non-coding RNA
LOD: Limit of detection
LTRs: Long terminal repeats
Lung-CLiP: Lung cancer likelihood in plasma
MCED: Multi-cancer early detection
MESA: Multimodal epigenetic sequencing analysis
miRNA: MicroRNA
ML: Machine learning
MRD: Minimal residual disease
MSLB: Multifeature sequencing-based liquid biopsy
mRNA: Messenger RNA
mtRNA: Mitochondrial RNA
NA: Not applicable
nanoRCS: Nanopore rolling circle amplification-enhanced consensus sequencing
NSCLC: Non-small cell lung cancer
NPC: Nasopharyngeal carcinoma
ONT: Oxford Nanopore Technologies
PALM-Seq: Polyadenylation ligation-mediated sequencing
piRNA: Piwi-interacting RNA
RAGs: Rare-abundance genes
RARE-seq: Random priming and affinity capture of cfRNA fragments for enrichment analysis by sequencing
RCA: Rolling circle amplification
RNA: Ribonucleic acid
RNases: Ribonucleases
RNA-seq: RNA sequencing
rRNA: Ribosomal RNA
SAA: Stepwise analytical alignment
SCLC: Small cell lung cancer
SCLCpheno-seq: Small cell lung cancer phenotyping sequencing
SINEs: Short interspersed nuclear elements
SMART-total: SMART-based total RNA sequencing
SNVs: Single-nucleotide variants
SPOT-MAS: Screening for the presence of tumor by methylation and size
sRNA: Small RNA
sRNAflow: Small RNA flow pipeline
sMETASeq: Small-RNA metagenomics by sequencing
SVM: Support vector machine
sWGS: Shallow whole-genome sequencing
sWMS: Shallow whole methylome sequencing
TAPS: TET-assisted pyridine borane sequencing
TEs: Transposable elements
TET2: Ten-eleven translocation 2
TF: Transcription factor
THEMIS: Thorough epigenetic marker integration solution
tRNA: Transfer RNA
UMI: Unique molecular identifier
WBC: White blood cell
WGBS: Whole-genome bisulfite sequencing
WGS: Whole-genome sequencing
