# Supplementary material for: Multifeature sequencing-based liquid biopsy for cancer diagnosis and monitoring
Source: Genome Med. 2026 Aug 1;18:116. doi: 10.1186/s13073-026-01739-2 (PMC13430740; doi:10.1186/s13073-026-01739-2)
Supplement: Supplementary file 1 — Additional file 1. [file 13073_2026_1739_MOESM1_ESM.docx]

**Table S1. Multimodal concepts in sequencing-based liquid biopsy analysis.**

| **Term** | **Description** | **Modalities** | **Method example** | **Features** |
| --- | --- | --- | --- | --- |
| Multifactor | Analysis of multiple variables within the same molecular feature class | Same data class | cfDNA-seq | CNAs at multiple loci |
| Multifeature | Extraction of multiple biological features from a single sequencing dataset | Same data set | cfDNA-seq | CNAs + fragmentomics |
| Multiomic | Integration of multiple molecular analyses from the same analyte | Multiple data sets | cfDNA- & RNA-seq | CNAs + gene expression |
| Multianalyte | Combined analysis of different circulating biological components | Multiple components | cfDNA- & CTC RNA-seq | CNAs + transcriptome |
| Multimodal | Integration of molecular, clinical, imaging, or physiological data types | Multiple data types | cfDNA- & RNA-seq & imaging | Genomic, transcriptomic,  and radiomic integration |
